# *Mycobacterium avium* subsp. *paratuberculosis* and Hashimoto’s thyroiditis: Is MAP the trigger?

**DOI:** 10.3389/fcimb.2022.972929

**Published:** 2022-09-20

**Authors:** Maedeh Moghadam, Ezzat Allah Ghaemi, Hamideh Akbari, Hadi Razavi Nikoo, Samin Zamani

**Affiliations:** ^1^ Department of Microbiology, School of Medicine, Golestan University of Medical Sciences, Gorgan, Iran; ^2^ Infectious Diseases Research Center, Golestan University of Medical Sciences, Gorgan, Iran; ^3^ Department of Endocrinology, Clinical Research Development Unit (CRDU), Sayad Shirazi hospital, Golestan University of Medical Sciences, Gorgan, Iran

**Keywords:** Hashimoto’s thyroiditis, *Mycobacterium avium* subspecies *paratuberculosis*, MAP3865c, IS900, map, elisa, Nested-PCR

## Abstract

Hashimoto’s thyroiditis (HT) is an autoimmune disorder of the thyroid gland that can cause hypothyroidism. As HT is a multifactorial disorder, activation of immune responses in genetically predisposed individuals exposed to some environmental factors can contribute to it. Microorganisms, as environmental factors, including *Mycobacterium avium* ssp. *paratuberculosis* (MAP) by molecular mimicry, can be important in this autoimmune disorder. This study aimed to investigate the association between MAP and HT. This case–control study included 110 participants consisting of 60 HT patients and 50 healthy controls (HCs). Blood samples were collected. Nested PCR of the IS900 gene determined the presence of MAP DNA. The enzyme-linked immunosorbent assay (ELISA) was designed to identify antibodies (Abs) against the MAP3865c epitope, which has a homologous sequence with ZnT8 in the sera. The demographic information of all participants was recorded. Anti-TG, anti-TPO, TSH, anemia, and ruminant exposure were higer in HT patients than in the HCs (*p* < 0.05). MAP IS900 was detected significantly more in the patients (46.6% consisting of 30, 8.3, and 8.3% in clinical, subclinical, and unknown) than in the HCs (14%). The sera showed a remarkable frequency of reactivity against MAP3865c in the patients (38.3%) in comparison to the HCs (10%) (*p* = 0.0001). Furthermore, a significantly higher rate of livestock contact and traditional dairy consumption was found in individuals with MAP or anti-MAP3865c Abs positive result (*p* < 0.05). This study suggests a possible link between MAP and HT. These findings indicated that MAP frequency was not statistically different in the severity of HT and its shift into the clinical and subclinical forms; therefore, it could be assumed that MAPs are the initiators of the process. The results imply on a possible zoonosis transmission route of MAP from livestock products to humans. Further research is needed to confirm these results in larger groups of HT patients.

## Introduction

Hashimoto’s thyroiditis (HT), known as chronic lymphocytic thyroiditis, is an autoimmune disorder involving chronic thyroid inflammation that can cause hypothyroidism and, rarely, hyperthyroidism (Ahmed et al., 2012; Sepasi et al., 2020). One of the characteristics of this disorder is the abundant exudation of B and T lymphocytes, leading to the destruction of the thyroid gland. The thyroid gland produces the hormones which help the body use energy and control different body areas (Ahmed et al., 2012). Both humoral and cellular immune responses are induced against thyroid antigens, including thyroid peroxidase (TPO) and thyroglobulin (TG) (Niegowska et al., 2015). Moreover, subclinical HT is a primary, mild form of HT. It can be diagnosed with an elevated serum thyroid-stimulating hormone (TSH) level despite an average serum-free thyroxin concentration. Subclinical and clinical HT may be related to metabolic disorders, cardiovascular risk, and quality of life (Cooper, 2001; Braverman and Cooper, 2012). Since HT is a multifactorial disease, immune responses are activated in genetically predisposed individuals exposed to environmental factors that contribute to the disease (Burek and Talor, 2009; Tomer and Huber, 2009). Microorganisms such as *Helicobacter pylori*, *Yersinia*, and *Mycobacterium avium* subspecies *paratuberculosis* (MAP) are important risk factors for HT (Çorapçioğlu et al., 2002; Aghili et al., 2013; Niegowska et al., 2015).

MAP is a facultative intracellular parasite that can multiply in the host cells of susceptible species, which has been suggested to stimulate Hashimoto’s disease (Sisto et al., 2010; Masala et al., 2014; Niegowska et al., 2015). The possible mechanism is molecular mimicry between the surface antigens of the bacteria, including MAP3865c, and the peptides of the follicular and parafollicular cells of the thyroid gland called ZnT8 (Wenzlau et al., 2007; Masala et al., 2014). The significant association of MAP with other autoimmune diseases such as Crohn’s disease (CD), type 1 diabetes, multiple sclerosis (MS), rheumatoid arthritis (RA(, lupus erythematosus, and sarcoidosis syndrome has been reported in some susceptible human hosts (Wenzlau et al., 2007; Masala et al., 2011; Naser et al., 2013; Kuenstner et al., 2015; Dow, 2016; Kuenstner et al., 2017; Bo et al., 2019). The molecular mimicry due to the high level of homology between MAP peptides and human homologs supported a possible mycobacterial role in triggering these autoimmune illnesses, too (Naser et al., 2013). Further studies indicated that anti-TNFα treatment for MAP-positive patients could result in advantageous states for MAP infection, which could be the reason for the poor response of some CD patients to the currently approved therapy (Qasem and Naser, 2018). On the other hand, controlled clinical trials explained that anti-MAP therapy could be helpful as an alternate treatment choice in CD patients, even in the absence of reliable MAP diagnostics (Kuenstner et al., 2015; Kuenstner et al., 2017; Qasem et al., 2020).

In Iran, some studies declared the presence of MAP in patients with CD, ulcerative colitis, and type 1 diabetes. The findings demonstrated a remarkable association between MAP and these autoimmune disorders (Shariati et al., 2016; Zamani et al., 2017; Zarei-Kordshouli et al., 2019; Amirizadehfard et al., 2020).

MAP is a causative agent of Johne’s disease (JD) and can involve the small intestine of livestock such as cattle and sheep, leading to chronic, contagious, and fatal infections (Sadeghi et al., 2020). In both clinical and subclinical forms of JD, animals can shed the bacteria through their feces. MAP can be present in pasteurized milk, infant formula, water, soil, meat, and domestic aerosols (Ozana et al., 2022). Since this bacterium is resistant to pasteurization and chlorination, the main transmission routes are water, food, and especially dairy products (Sechi and Dow, 2015; Ozana et al., 2022). JD is endemic in Iran, and some studies reported MAP in cattle, sheep, and goats in different parts of Iran. In a survey conducted in the north of Iran, the prevalence of MAP in milk samples ranged from 4.2 to 7.7% (Sadati et al., 2012). However, there is no information about the prevalence of this bacterium in our province. Traditional agriculture and livestock are common in Northeast Iran. Besides this, there are limited data about the trend of Hashimoto’s prevalence in the region. However, endocrinologists declare that the prevalence of the disease has increased in recent years (Sepasi et al., 2020). This study aimed to determine the association between MAP and HT by detecting the bacterium gene and anti-MAP antibodies (Abs) in HT patients and controls using indirect enzyme-linked immunosorbent assay (ELISA) and nested PCR. Further risk factors that contributed to HT and MAP were also investigated.

## Materials and methods

### Study population, clinical characteristics, and sample preparation

The clinical samples of this study were 110 blood samples. Sixty individuals were HT confirmed cases according to the endocrinologist’s order, and 50 were healthy controls. The Ethics Committee of Golestan University of Medical Sciences approved the protocol of this study (project number: 110952 and approval ID: IR.GOUMS.REC.1398.250). Data on the demographic and clinical characteristics of patients such as age, gender, weight, history of any disease, and dairy consumption were collected from the patients’ electronic records. The endocrinologist approved the HT cases because of hypothyroidism symptoms, including serum TSH >4.12 mIU/L and increased antibody titers of TG or TPO. The raised serum TSH levels combined with normal levels of serum-free thyroid hormones such as triiodothyronine (T4) and free thyroxin (T3) hormones were defined as subclinical HT (Cooper, 2001; Braverman and Cooper, 2012). Individuals with any known autoimmune disease mentioned in the questionnaire, such as RA, diabetes, MS, inflammatory bowel disease (IBD), and other known autoimmune diseases, were excluded from the study. The control group was comprised of persons without a history of thyroid disorder or known autoimmune diseases such as RA, diabetes, MS, and IBD. Five milliliters of whole blood samples was collected from both the patients and the control group. The specimens were then transported on ice to the microbiology department at Golestan University of Medical Sciences, Gorgan, Iran. The sera were separated and stored at 4–8°C for short-term storage or at –70°C for the longer term. The blood is used to extract peripheral blood mononuclear cells (PBMCs). According to the manufactured instruction, a standard extraction protocol with a DNA extraction kit (Gene All Biotechnology, South Korea) was utilized for PBMCs to extract the bacterial DNA. The sera were used for further ELISA.

### Detection of anti-MAP3865c_125–133_ Abs by In-house indirect ELISA

Sera were assayed for IgG-specific MAP peptides by endpoint dilution ELISAs. A 96-well flat-bottomed plate (Nunc, Denmark) was coated with MAP3865c (amino acid residues 125–133; MIAVALAGL, 2 μg/ml) in coating buffer (Na_2_CO_3_ and NaHCO_3_, pH 7.4) by overnight incubation at 4°C. The plates were washed three times with phosphate-buffered saline (PBS) containing 0.05% Tween 20 (PBST) and blocked by adding 100 μl of blocking solution (PBS-T containing 2% gelatin) to each well at 37°C for 2 h. After washing, serial twofold dilutions of sera in PBS were added to the plates and incubated on the shaker at 37°C for 2 h. The same dilutions of the sera were tested on uncoated wells to determine nonspecific binding. Following washing, horseradish peroxidase-conjugated goat anti-human IgG antibody (RaziBiotec AP8036) at 1/5,000 dilution was added. After 1.5 h of incubation on the shaker at 37°C and subsequent washing, 100 μl 3,3′,5,5′-tetramethyl benzidine substrate solution (Sigma T4444) was added to the wells as substrate, and the plates were incubated at room temperature for 30 min. Finally, a stop solution (2 N H_2_SO_4_, 50 μl) was added to the wells, and absorbance was measured at 450 nm using an ELISA plate reader. Endpoint titers were defined as the reciprocal of the highest serum dilution giving an absorbance value greater than the average absorbance of a negative control human plus three times the standard deviation (SD). They were expressed as the group means ± SD for each sample.

### Nested polymerase chain reaction

A nested PCR was carried out to investigate the presence of bacterial DNA in both groups. All of the samples were tested by nested PCR with specific primers for regions of the IS900. The appropriate forward and reverse primers for the first and the second amplifications are presented in **Table 1**. The PCR products were electrophoresed using 2% agarose gel, and the sizes of the amplicon determined the second product. In the first round, the thermal cycle conditions were as follows: one cycle at 95°C for 5 min, 40 cycles including three steps at 95°C for 15 s, 58°C for 30 s, and 72°C for 30s, and a final cycle at 72°C for 10 min. The second round was performed as described for the first round with some changes in annealing temperature and cycles. The PCR products were sequenced (Pishgam Inc., Tehran, Iran) to validate the bacterium gene further.

**Table 1:** Internal and external primers.

Number	Gene	Primer	Amplicon size (bp)	Reference
1	L	F: CTTTCTTGAAGGGTGTTCGG	402	(Bull et al., 2003)
		R: ACGTGACCTCGCCTCCAT		
2	AV	F: ATGTGGTTGCTGTGTTGGATG G	298	
		R: CCGCCGCAATCAACTCCAG		

### Statistical analysis

The data were statistically analyzed by the software SPSS, version 19.0. Differences between the groups according to the variables were analyzed using the appropriate tests. Categorical variables were stated as percentage and frequency, and numerical variables were expressed as mean ± SD according to their distribution pattern. According to their distributions, we used Student’s *t*-test or the Mann–Whitney *U*-test to compare the numerical variables between the two groups and Fisher’s exact test and chi-square test to compare the categorical variables. A *p*-value <0.05 was considered statistically significant.

In this design, the odds ratio (OR) is a consistent estimator of the rate ratio of HT when exposed to MAP vs. unexposed individuals. The OR was evaluated using an Excel spreadsheet described by Newcombe et al. (Newcombe, 2016). A *p*-value <0.05 was considered statistically significant.

## Results

### Characteristics of clinical specimens and demographic data

The present study included 110 participants, of which 60 were HT confirmed cases (with a mean age of 38.1 ± 1.2 years), and 50 were healthy persons (with a mean age of 38.6 ± 1.3 years). The study of clinical symptoms showed that 36 and 14 patients had clinical and subclinical pictures, respectively. Furthermore, the clinical and subclinical data of 10 patients were missing. **Table 2** summarizes the demographic (age and sex) and laboratory parameters of the participants included in this study. The anti-TG, anti-TPO, and TSH levels in the patients were 672 ± 364 IU/ml, 564 ± 444.6 IU/ml, and 15.9 ± 8.3 mIU/L, respectively, which were more than those of the controls (14.6 ± 13.8 IU/ml, 4.1 ± 6.8 IU/ml, 2.5 ± 1.5 mIU/L, respectively) (*P* < 0.05). In addition, the HT patients had more ruminant exposure than the controls (70% in HT vs. 30% in the controls, *P* = 0.01). About 88 and 62.5% of oily and dry skin types were reported in the HT groups, which were significantly more than the controls (*P* = 0.0001). Consumption of traditional dairy was more frequent in the patients (82.6%) than in the controls (17.4%) (*P* = 0.008). Anemia was diagnosed in 83.3 and 16.7% of HT patients and the controls, respectively (*P* = 0.0001). These differences were statistically significant.

**Table 2:** Demographic, clinical manifestation and laboratory parameters of the participants.

Variable		Hashimoto thyroiditis	Controls	P-value
Female, n (%)*		54 (90%)	42 (84%)	0.2
Mean age (years)		38.1±1.2	38.6±1.3	0.8
Mean weight (Kg)		7.3±65.9	13.7±70.8	0.02
Anti-TG (IU/mL)		364±672	13.8±14.6	0.0001
TSH (mIU/L)		15.9±8.3	2.5±1.5	0.01
Anti-TPO (IU/mL)		564±444.6	4.1±6.8	0.0001
T3 value (ng/dL)		103.4±53.6	94.1±27.4	0.6
T4 value (ng/dL)		6.5±2.1	6.3±1.1	0.3
Skin, n (%)*	Dry	22 (88%)	12.8 (12%)	
	Oily	10 (62.5%)	6 (37.5%)	0.0001
	Normal	24 (39.3%)	37 (60.7%)	
Ruminant’s exposure, n (%)*	Yes	14 (70%)	6 (30%)	
	No	29 (29.7%)	44 (60.3%)	0.01
Anemia, n (%)*	Yes	20 (83.3%)	4 (16.7%)	
	No	26 (61.2%)	41 (38.8%)	0.0001
Dairy consumption, n (%)*	Pasteurized	15 (41.7%)	21 (58.3%)	
	Traditional	19 (82.6%)	4 (17.4%)	0.008
	Both	12 (52.12%)	11 (47.8%)	
Heart problems, n (%)*	Yes	5 (83.3%)	1 (16.7%)	
	No	36 (81.8%)	8 (18.2%)	0.7
Depression, n (%)*	Yes	5 (71.4%)	2 (28.6%)	
	No	35 (87.5%)	5 (15.5%)	0.2

*Data are presented as n (%), (mean ± SD), according to the variable. “n” indicates the number of patients. Anti-TPO, Anti-thyroid Peroxidase Antibody; Anti- TG, Anti-thyroglobulin antibody; TSH, Thyroid Stimulating Hormone. *variables.

### Identification of MAP in HT-positive and healthy persons

According to our results, 35 (32%) samples were positive MAP based on the molecular test. The nested PCR analysis was positive in 28 of 60 HT-positive patients (46.6%) for the presence of the IS900 gene. MAP frequency was significantly higher in the HT-positive group compared to the controls (14%) (*p* < 0.00001). Among patients with HT who were positive for the bacterial gene, 64.3% (18/28) were in the clinical phase, and 17.9% (5/28) were in the subclinical phase. Clinical and subclinical information was missing for 17.9% (5/28) of HT patients who were positive for the bacterial gene.

The positive PCR products were validated for MAP by sequencing. The data is available in NCBI databases with the following accession numbers: MW650835, MW714386, MW714387, MW714388, and MW714389.

### Analysis of serum antibody against MAP3865c_125–133_ peptide

**Table 3** indicates the nested PCR results for MAP IS900 gene, ELISA for anti-MAP3865c_125–133_ Abs, and nested PCR- or ELISA-positive results in HT patients and controls. The nested PCR- or ELISA-positive individuals in **Table 3** refer to those with at least one positive result for the presence of the IS900 gene or anti-MAP3865c_125–133_ Ab. The antibody against MAP3865c_125–133_ peptide was 38.3% (23/60) in the patients and 10% (5/50) in the controls; this difference was statistically significant (*p* < 0.001). Among HT patients who were positive for anti-MAP3865c_125–133_ Abs, 60.8% (14/23) were in the clinical phase, 21.7% (5/23) were in the subclinical phase, and 17.4% (4/23) of them were unknown. Further results demonstrated that 60% (36/60) of the HT patients compared to 20% (10/50) controls have at least IS900 gene or anti-MAP3865c_125–133_ Abs, which is statistically significant.

**Table 3:** Nested-PCR results of MAP IS900 and ELISA results for MAP3865c_125-133_ in HT patients and controls.

Groups		PCR	ELISA	^*^PCR or ELISA
		MAP IS900 +	Anti-MAP3865c Abs +	MAP IS900 or Anti-MAP3865c Abs +
** **	^**^Clinical	18 (30%)	14 (23.3%)	19 (31.6%)
** **	Subclinical	5 (8.3%)	5 (8.3%)	9 (15%)
**HT**	Unknown	5 (8.3%)	4 (6.6%)	8 (13.3%)
	Total	28 (46.6%)	23 (38.3%)	36 (60%)
**Control**		7 (14%)	5 (10%)	10 (20%)
***P-* value**		0.0001		0.0001

^*^Nested-PCR or ELISA positive refers to individuals with at least one positive result; the presence of the IS900 gene or anti-MAP3865c_125-133_ Ab.

^**^No significant differences were observed in PCR, MAP Ab, and PCR or ELISA positive results between clinical and subclinical (p-value > 0.05).

**Figure 1:**
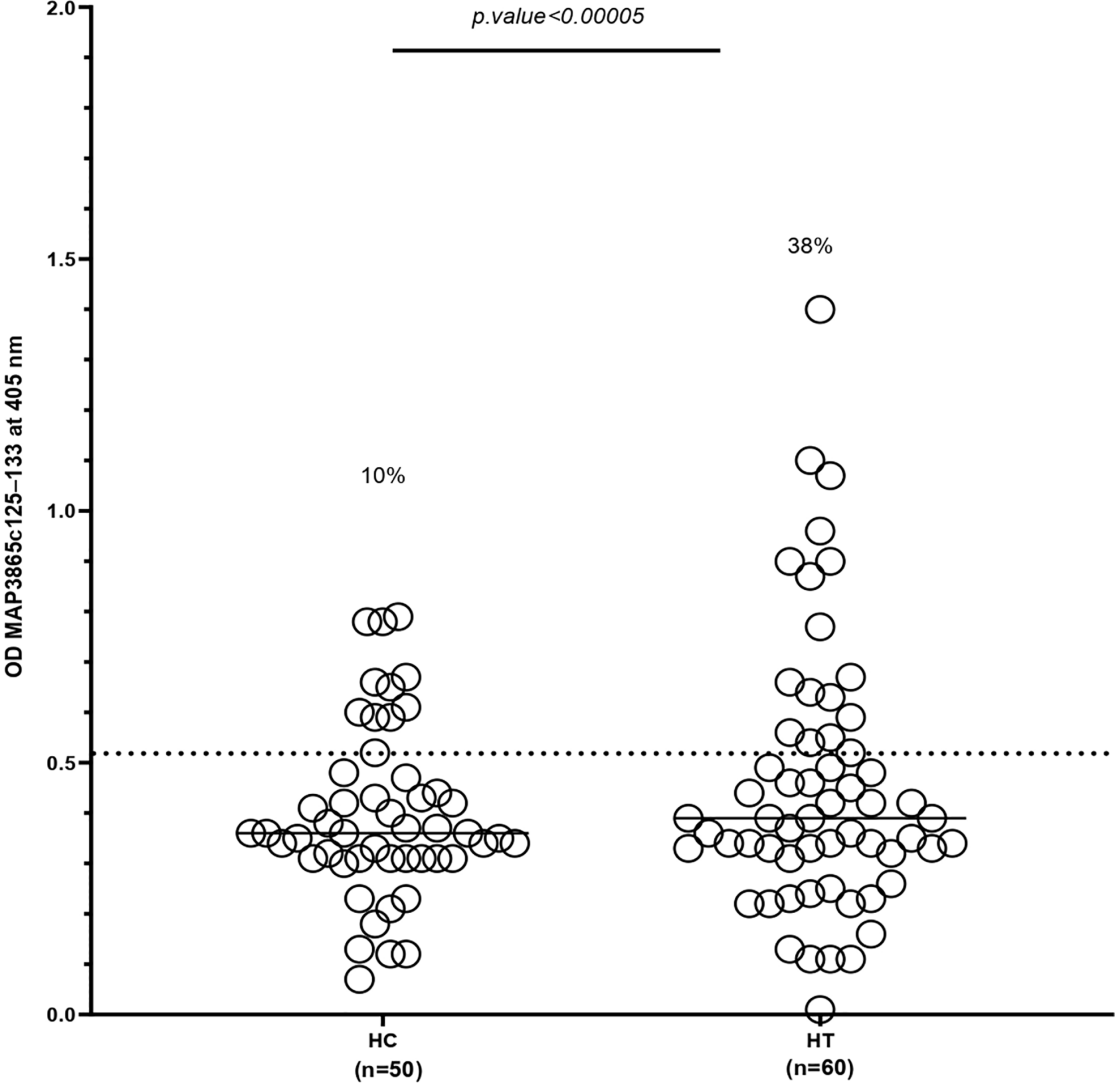
Antibody levels against MAP3865c_125–133_ peptide in HT patients and HCs. The sera were examined in duplicate for their reactivity plate-coated MAP3865c_125–133_ peptide. The x-axis represents the group of patients and controls, and the y-axis represents the OD for the MAP3865c_125–133_ Abs. The dotted lines display thresholds of positivity relative to each assay (cutoff = 0.51). The percentage of Abs-positive is reported on top of each distribution. *P*-values (95% CI) are indicated above the graphs. HC, healthy control; HT, Hashimoto’s thyroids. The cutoff value was based on the receiver operating characteristic curve with 95% confidence interval.

The distribution of antibodies against the MAP3865c_125–133_ peptide in the HT group and the controls is exhibited in **Figure 1**. The x-axis indicated the group of the patients and controls, and the y-axis displayed the OD for the MAP3865c_125–133_ Abs. The dotted lines showed the thresholds of positivity (cutoff = 0.51) to each assay.

**Table 4** shows the distribution of at least one positive result for the presence of the IS900 gene or anti-MAP3865c_125–133_ Ab in the patients and controls according to demographic factors such as gender, age, weight, disease period, animal contacts, types of milk consumption, and titers of anti-TPO, anti-TG, and TSH levels. The results presented no significant relationship between gender, age, weight, disease period, titers of anti-TPO, anti-TG, and TSH, and the presence of anti-MAP3865c_125–133_ Abs or IS900 gene (*p* > 0.05). The results demonstrated that contact with livestock was higher in the individuals who had at least one positive result than in those they did not. In addition, the consumption of traditional milk in the group with positive results of ELISA or nested PCR was more than in the groups with negative results (*p* < 0.05). Moreover, we analyzed the occupation of the patients and controls, and the results showed no significant relationship between it and nested PCR or ELISA positivity (*p* > 0.05). Similarly, meat consumption was almost the same in both groups (*p* > 0.05).

**Table 4:** Statistical results of demographical and laboratory tests data among MAP positive in the patients and control group.

Variable		MAP IS900 PCR + or Anti-MAP3865c antibody +		
		Hashimoto thyroiditis	Control	Significance
		36 (60 %)	10 (20%)	
Female, n		32	8	Ns
Mean age (years)		36.8±12.09	29.5±8.3	Ns
Mean weight (Kg)		72.7±15.8	65.50±8.1	Ns
Anti-TG (IU/mL)		264.2±287.3	13.34±12.21	Ns
Anti-TPO (IU/mL)		480.1±568.01	8.03±4.43	Ns
TSH (mIU/L)		4.59±3.02	3.21±1.6	Ns
T3 (ng/dL)		109.7±56.6	102.33±30.54	Ns
T4 (ng/dL)		6.8±1.6	5.86±1.02	Ns
Animal contact, n (%)	Yes	22 (78%)	5 (50%)	
	No	10 (38%)	3 (9%)	< 0.05
*Milk consumption, n (%)	Traditional milk	17 (80%)	4 (66%)	
	Pasteurized milk	15 (45%)	4 (11%)	< 0.05

*For 11 cases, no information was available on the type of milk consumed. Data are presented as n (%), (mean ± SD), according to the variable. “n” indicates the number of patients; Anti-TPO, Anti-thyroid Peroxidase Antibody; Anti- TG, Anti-thyroglobulin antibody; TSH, Thyroid Stimulating Hormone. Ns, Not significance.

### Odds ratio analysis

In **Table 5**, the results demonstrated that individuals with positive MAP results based on nested PCR were 5.375 times more likely to develop Hashimoto’s disease than those without the bacterium (OR = 5.375, 95% CI: 2.08–13.84) (*p*-value = 0.0005).

**Table 5:** The OR of HT when exposed with MAP vs. unexposed individuals.

	OR	Standard error	*Z*	*p*-value	95% CI
**MAP positive**	5.375	2.5	3.48	0.0005	2.08–13.84

OR, odds ratio; 95% CI, 95% confidence interval.

## Discussion

HT is an autoimmune thyroid illness in which the human immune system perceives and reacts to the thyroid gland (Cooper, 2001; Braverman and Cooper, 2012). The prevalence and the incidence of HT have increased significantly in recent years (Ralli et al., 2018; Sepasi et al., 2020). The mechanism of this disorder has not been well known, and different genetics, epigenetics, and lifestyle factors can play roles in disease development (Ralli et al., 2018). The critical environmental factors are microorganisms, including different bacteria and viruses such as Epstein–Barr virus, Cytomegalovirus, hepatitis C virus, Enterovirus, Flavivirus, *Yersinia enterocolitica*, *Helicobacter pylori*, *Rickettsia*, *Coxiella burnetii*, *Staphylococcus*, *Streptococcus*, and MAP (Ralli et al., 2018; Machała et al., 2019).

MAP is the causative agent of JD in ruminants, whose role has been investigated as one of the initiators of various autoimmune diseases in susceptible human hosts (Ekundayo and Okoh, 2020). Some previous studies described the association of MAP with conditions such as CD, type 1 diabetes, RA, MS, lupus erythematosus, and Blue syndrome (Naser et al., 2004; Masala et al., 2011; Naser et al., 2013; Kuenstner et al., 2015; Niegowska et al., 2015; Sechi and Dow, 2015; Dow, 2016; Kuenstner et al., 2017; Zamani et al., 2017; Bo et al., 2019; Ekundayo and Okoh, 2020; Kuenstner et al., 2020). The culture and PCR-based detection of MAP from the blood of these autoimmune patients and the supportive role of anti-MAP therapy provide helpful evidence for the zoonotic feature of MAP as Kuenstner et al. explained in their study (Naser et al., 2010; Kuenstner et al., 2015; Kuenstner et al., 2020). Although its possible role in the onset of various autoimmune diseases has been discussed, globally limited information on this association with HT disease has been presented. The mechanism for this link is molecular mimicry between the surface antigens of MAP, such as MAP3865c, and the peptides of the thyroid gland’s follicular and parafollicular cells, such as ZnT8 (Masala et al., 2014; Niegowska et al., 2015).

IS900 is a distinctive marker of MAP detection. We used sensitive and specific nested PCR to detect the MAP-associated IS900 gene. The results showed that MAP was significantly more abundant in the patients than in the controls (*p* < 0.00001). An analysis of the results indicated that the risk of Hashimoto’s disease is about five times higher in patients with positive molecular results than in individuals with negative results, which means a possible role of MAP in HT (*p* = 0.0005). The frequency of MAP was likewise higher in the clinical (50%) than in the subclinical (35.7%) HT groups, which indicated the importance of a recent MAP infection in the development and the severity of the disease. Subclinical cases are often characterized by asymptomatic or vague symptoms leading to the diagnosis being missed; after a while, they frequently progress to the clinical patients (Khan et al., 2017). The other studies demonstrated that the human’s leading MAP colonization site is the terminal ileum. However, we searched for MAP in the blood, and the negative results for the MAP gene in some HT patients may be due to this matter (To et al., 2020). Moreover, due to the multi-factor nature of HT, different microbes and other factors can play a role in disease development (Machała et al., 2019).

Limited data from studies investigating the relationship between the MAP IS900 gene and HT (Sisto et al., 2010; D’Amore et al., 2010; Gupta et al., 2017) have been reported. In a case report by Sisto *et al.*, the IS900 gene was identified in a patient with HT (Sisto et al., 2010). The other studies also detected MAP IS900 in HT patients (D’Amore et al., 2010; Gupta et al., 2017). In addition, Gupta *et al.* indicated that the Taqman probe and SYBR Green Real-time PCR methods were more sensitive and highly specific than PCR for detecting a MAP infection (Gupta et al., 2017).

A previous infection with MAP may trigger HT syndrome. Therefore, in patients with a long history of HT, it is better to look for antibodies against MAP peptides or homologous peptides than the bacterial genome. In this study, the bacterial peptide MAP3865c_125–133_ was used for indirect ELISA (Masala et al., 2014; Niegowska et al., 2015). Antibodies against this peptide do not precisely confirm a MAP infection because of a homologous peptide such as ZnT8 in humans; therefore, the ELISA results may not be specific for MAP. The rate of positivity of anti-MAP3865c Abs was significantly higher in patients with HT (38.3%) than in the controls (10%) (*p* < 0.001). Limited data from studies investigating the relationship between MAP Abs and HT have been reported. Niegowska et al. identified antibodies against proinsulin and MAP-derived homologous epitopes, including MAP1,4αgbp_157-173_/PI_64-80_, in HT patients and controls. Their results indicated higher levels of Abs in HT patients than in the controls (*p* < 0.0003 and *p* < 0.002, respectively), which were statistically significant. Additionally, they investigated the homologous peptides of MAP2404c70-85/PI46-61. The results showed a high prevalence of Abs against these peptides in the patients, which was not statistically significant (Niegowska et al., 2015). In the study of Masala *et al.*, two pairs of homologous peptides consisting of MAP3865c_125–133_/ZnT8_178–186_ and MAP3865c_133–141_/ZnT8_186–194_ were studied. These results indicated the presence of a link between MAP seroreactivity and HT (Masala et al., 2014).

Our result indicated a higher frequency of anti-MAP3865c antibodies than in other studies (Masala et al., 2014; Niegowska et al., 2015). Undoubtedly, different environmental and genetic conditions in diverse populations could be considered. Our population lives in the northeast of Iran and near the Caspian Sea in Golestan Province, where traditional animal husbandry and agriculture rates are common. Since spreading from livestock to the environment is one of the most critical routes of transmitting MAP, it may justify people’s exposure to the bacterium. For several decades, paratuberculosis and meat, milk, and cheese contamination from cattle, sheep, and goats have spread globally, and humans may be threatened. In addition, it might present alive MAP or decay products in pasteurized milk and infant formula (Ozana et al., 2022). Some studies confirmed that the prevalence of MAP is relatively considerable in cattle, sheep, and goats in different parts of Iran** **despite no data about MAP prevalence in Golestan Province (Sadati et al., 2012; Anzabi and Khaki, 2017; Sadeghi et al., 2020; Pourmahdi Borujeni et al., 2021). In the study of Sadeghi *et al.*, MAPs were detected in 39 and 4.9% of 544 pasteurized milk samples by nested PCR and the culture method, respectively (Sadeghi et al., 2020). In addition, other studies demonstrated a relatively high occurrence of MAP in pasteurized milk by PCR-based methods. Regarding the possible etiological role of MAP in developing autoimmune disorders, this high prevalence of MAP can be considered a prominent concern for public health (Anzabi and Khaki, 2017).

Moreover, the nested PCR results were not consistent with indirect ELISA. This discrepancy led us to speculate that antibodies might have been produced against other peptides, which have not been investigated in the present study. On the other hand, MAP infection may have occurred in the distant past, whereas the antibodies remained over time. We should likewise consider that due to the homologous sequence of this peptide in humans, ELISA results could not be specific for MAP detection. Further studies are obviously needed to test Abs against homologous peptides. The other limitation of this study was the small sample size. It sounds statistically helpful and valuable to collect a large number of samples from HT and healthy individuals.

In addition, the mean duration of HT was higher in the group with positive results of anti-MAP Abs, which was statistically significant (*P* = 0.01). Therefore, we could conclude that these individuals were possibly in the chronic phase of the presence of MAP and HT.

About 14% of the healthy controls had MAP-positive results based on nested PCR. The positivity of MAP in the control group raises the question of whether these individuals may also develop HT in the future. Understanding this issue requires a longer follow-up of this group of people and designing a cohort study that we suggest to be considered in future research.

## Conclusion

In conclusion, the present study showed a statistically significant relationship between MAP and HT, and this bacterium could be one of the risk factors for HT. MAP detection in some controls indicated that MAP might be present in the region. Therefore, meat and dairy processing units and animal health systems should consider preventive and control procedures. These results also necessitate a study on the abundance of MAP in animal communities, especially JD, in the region. In addition, our results showed that the concurrence of the IS900 gene and anti-MAP Abs existence was significantly associated with HT. However, these data required further investigation of Abs against homologous peptides. Interestingly, the present findings indicated that the MAP gene or anti-MAP3865c Abs positivity was not statistically different in the severity of HT and its shift into clinical and subclinical forms. Therefore, it could be assumed that MAPs are the initiators of the process that leads to HT, and the other stages are independent. However, we should consider the alternative hypothesis that MAP may colonize more readily once the disease has been established. Understanding this issue requires further studies, especially in animal models.

## Data availability statement

The original contributions presented in the study are included in the article. Further inquiries can be directed to the corresponding author.

## Ethics statement

The studies involving human participants were reviewed and approved by the Ethics Committee of Golestan University of Medical Sciences. (Approval ID: IR.GOUMS.REC.1398.250). The patients/participants provided theirwritten informed consent to participate in this study.

## Author contributions

MM is the first author who performed all laboratory experiments, collected and analyzed data, and drafted the manuscript. EG participated in the coordination and advised in all parts of the study. HA was the endocrinologist who diagnosed and validated patients with Hashimoto’s thyroiditis and provided the specimens from all cases. HRN participated in the design and setup of the ELISA test. SZ participated in the study design and coordination and supervised all study parts. All authors contributed to the article and approved the submitted version.

## Funding

Golestan University of Medical Sciences supported this study (project number: 110952).

## Acknowledgments

The data of this article was related to the M. Sc. thesis of the first author at Golestan University of Medical Sciences. The authors would like to thank the personnel of the Department of Microbiology, Golestan University of Medical Sciences, Gorgan, Iran, for the technical services.

## Conflict of interest

The authors declare that the research was conducted in the absence of any commercial or financial relationships that could be construed as a potential conflict of interest.

## Publisher’s note

All claims expressed in this article are solely those of the authors and do not necessarily represent those of their affiliated organizations, or those of the publisher, the editors and the reviewers. Any product that may be evaluated in this article, or claim that may be made by its manufacturer, is not guaranteed or endorsed by the publisher.
